# Supplementary material for: Improving Conformational Ensembles of Folded Proteins in Go̅Martini
Source: J Chem Theory Comput. 2026 Feb 25;22(5):2369–79. doi: 10.1021/acs.jctc.5c01816 (PMC12980703; doi:10.1021/acs.jctc.5c01816)
Supplement: Supplementary file 1 [file ct5c01816_si_001.pdf]

Supporting Information

Improving conformational ensembles of folded proteins in GōMartini

Maksim Kalutskii[1,#], Carter J. Wilson[2,#], Helmut Grubmüller[1] and Maxim Igaev[1*]

[1]Department of Theoretical and Computational Biophysics, Max Planck Institute for Multidisciplinary Sciences, 37077 Göttingen, Germany

[2]Computational Biomolecular Dynamics Group, Max Planck Institute for Multidisciplinary Sciences, 37077 Göttingen, Germany

[#]Authors contributed equally.

[*]E-mail: migaev@mpinat.mpg.de

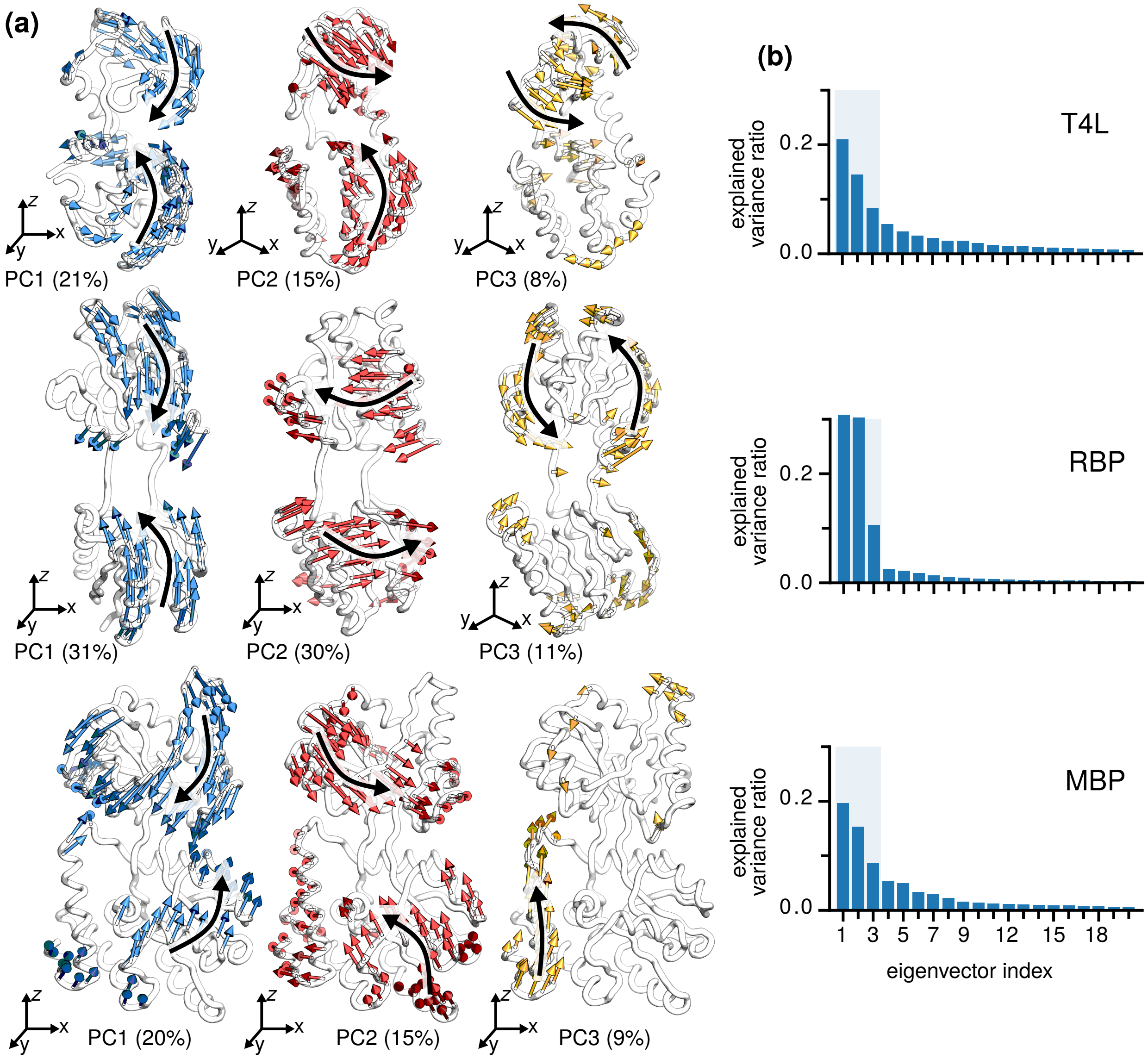

Figure S1: **(a)** Projected motions of the first three principal components. **(b)** Explained variance of the first twenty components.

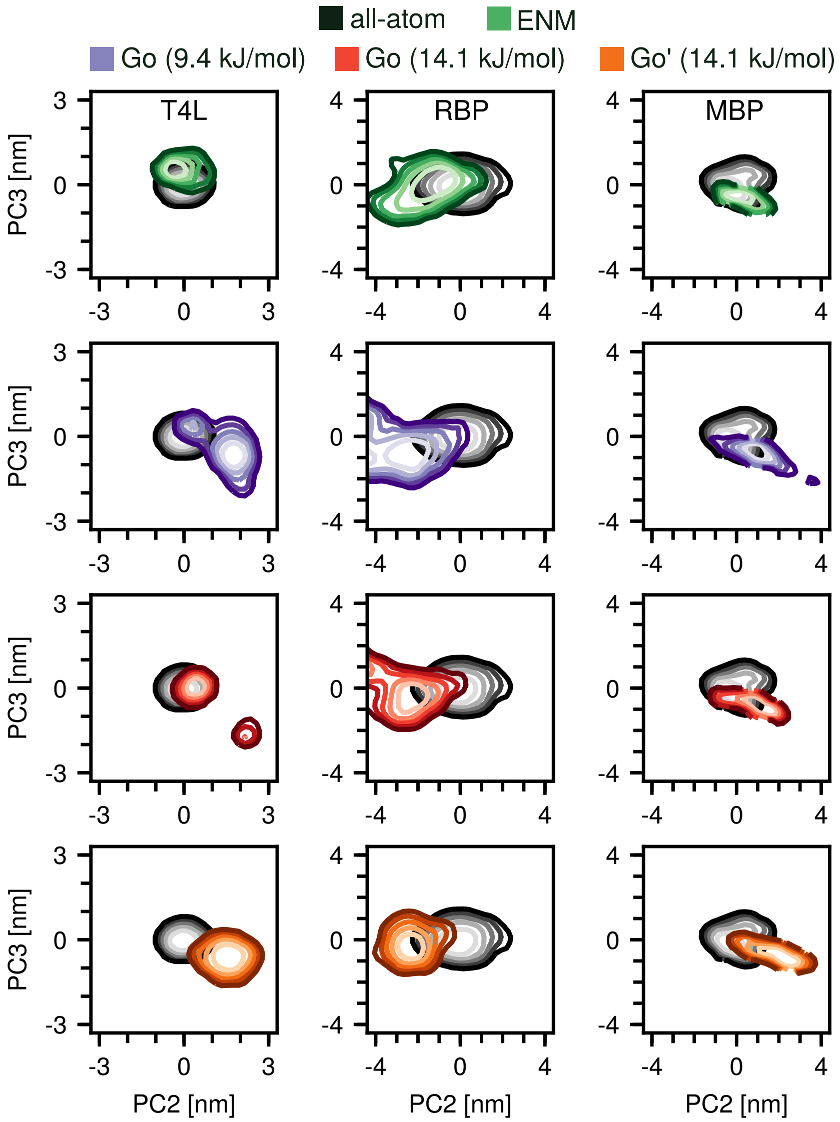

Figure S2: All-atom MD and CG trajectories projected onto the essential subspace of the MD ensembles (PC2 and PC3). Each column corresponds to a different protein, and each row corresponds to a different CG setup.

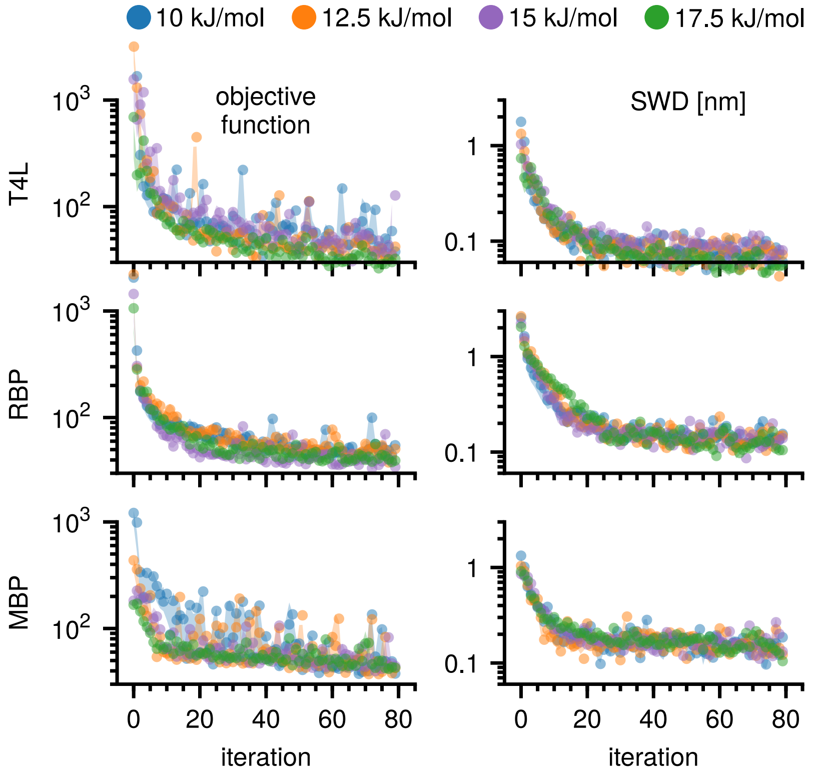

Figure S3: Convergence behavior for optimizations started with different uniform network strengths.

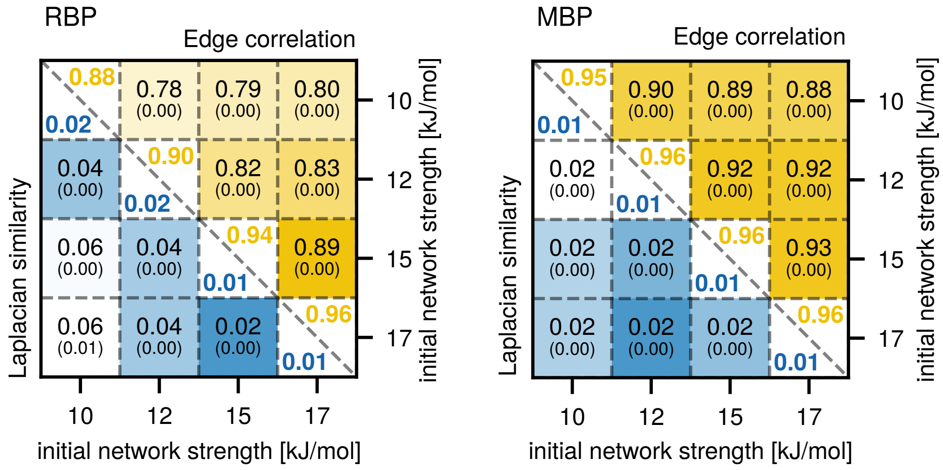

Figure S4: Correlation properties of Gō networks initialized from different uniform strengths. Standard errors are indicated in brackets. Numbers on the diagonal correspond to intra-replica metrics.

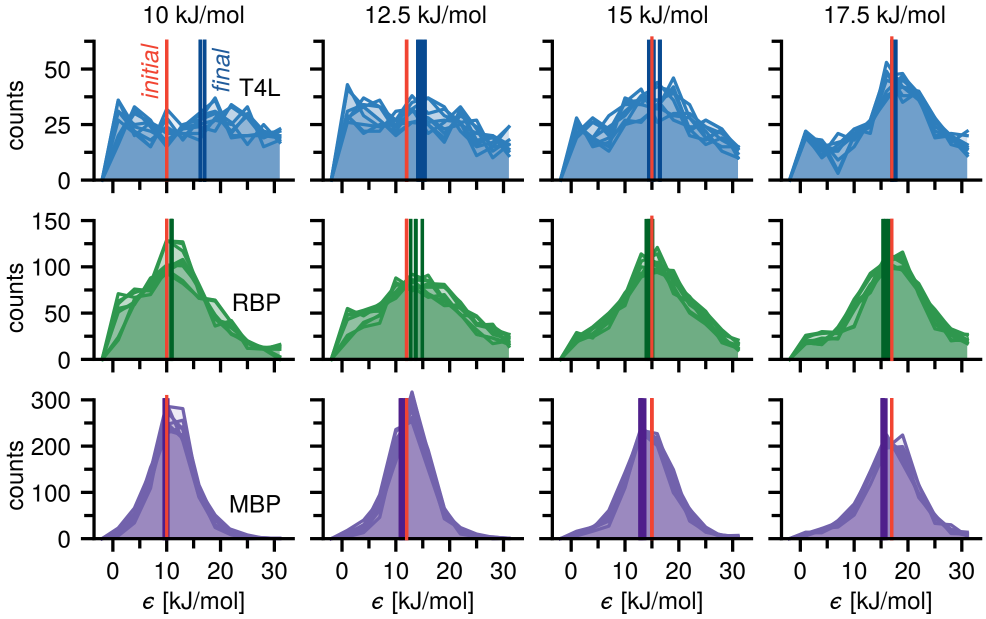

Figure S5: Distribution of the optimized Gō network edge weights as a function of the starting network strength. Initial and final averages are indicated with vertical lines.

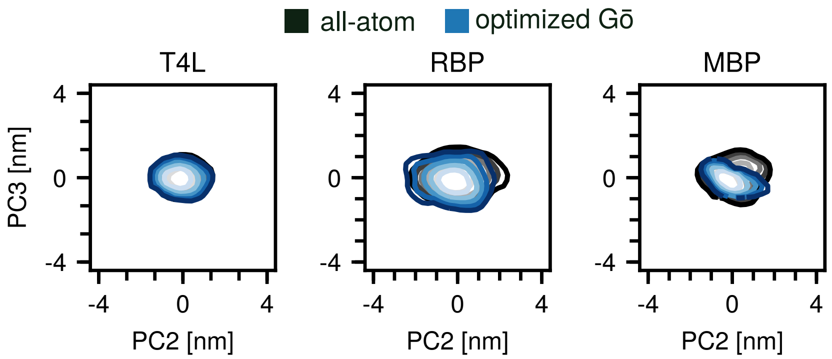

Figure S6: All-atom MD and optimized CG trajectories projected onto the essential subspace of the MD ensembles (PC2 and PC3). Each column corresponds to a different protein.

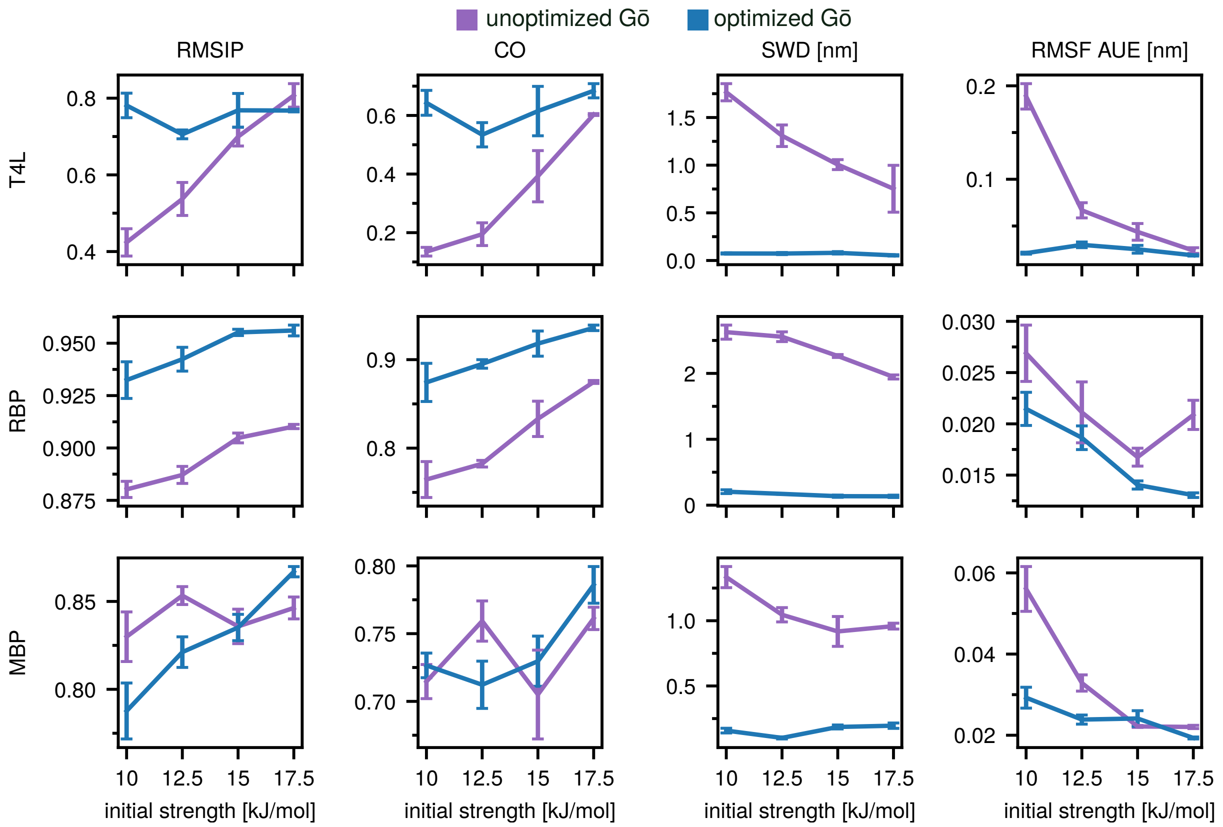

Figure S7: Impact of the initial Gō network strength on both unoptimized (purple) and optimized (blue) structural metrics for T4L (upper row), RBP (middle row) and MBP (bottom row). Error bars correspond to three independent repeats.

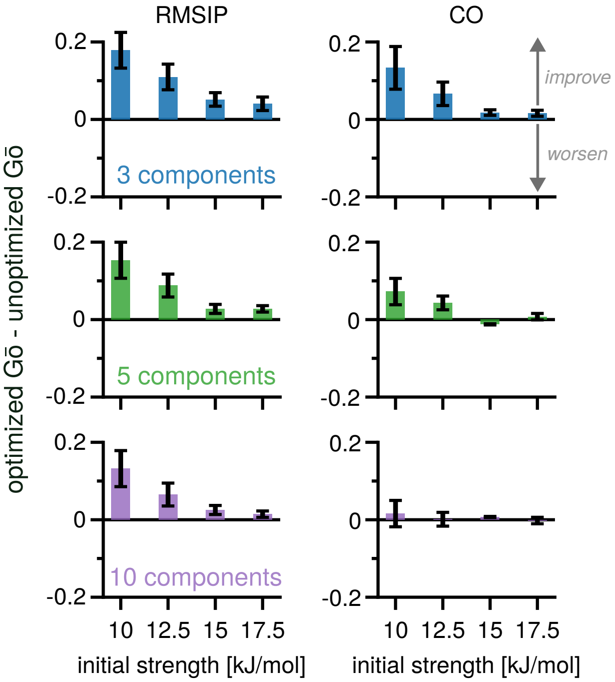

Figure S8: Change in the RMSIP and CO between the unoptimized and optimized Gō networks averaged over proteins and replicates. 3, 5, and 10 principal components were considered for the calculations. Positive values indicate improved agreement with the atomistic reference over unoptimized while negative values indicate worse agreement.
